# Renal pseudoaneurysm after core-needle biopsy of renal allograft successfully managed with superselective embolization

**DOI:** 10.1590/S1677-5538.IBJU.2014.0315

**Published:** 2016

**Authors:** Ioannis M. Antonopoulos, Kleiton Gabriel Ribeiro Yamaçake, Bruno C. Tiseo, Francisco C. Carnevale, Enio Z. Junior, William C. Nahas

**Affiliations:** 1 Divisão de Urologia, Hospital das Clinicas, Universidade de São Paulo, São Paulo, SP, Brasil

## INTRODUCTION

Renal biopsy of the allograft is important to evaluate renal dysfunction (1). Rare complications like pseudoaneurysm (PA) can develop and could lead to life-threatening bleeding (2, 3). It can be safely and effectively managed by endovascular embolization yielding good renal function in the long term follow-up (4, 5). We describe a PA of a kidney transplant (KTX) associated with arteriovenous fistula (AVF) at the site of a core needle percutaneous biopsy (CNPB).

## CASE DESCRIPTION

A 39-year old woman with nephrosclerosis and in hemodialysis for the last 3 years received a KTX from a deceased 20-year old male donor that had a cranio-cerebral trauma. The vascular anastomoses were performed at the right iliac vessels in an end-to-side fashion after 23 hours of cold ischemia.

**Figure 1:**
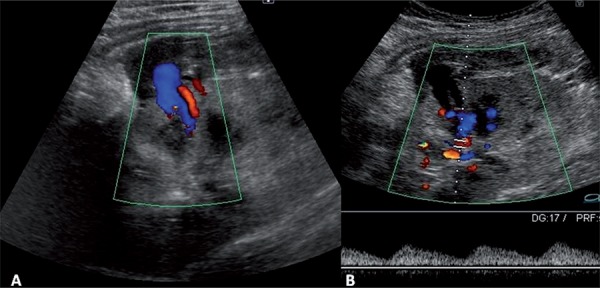
A-Doppler ultrasound with reverse diastole in interlobular artery and lesion suggestive of pseudoaneurysm at the middle pole of the kidney. Cystic formation which implies the renal parenchyma toward the collection, that measured about 1.2cm. This structure has bidirectional blood flow, suggestive of a pseudoaneurysm. B–Pulsatile flow in the vein suggestive of AVF.

**Figure 2:**
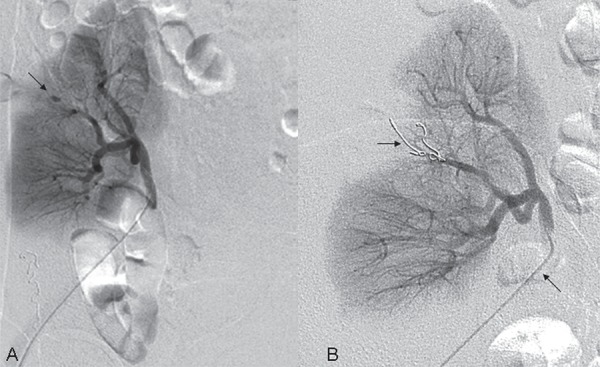
Renal transplanted arteriography: (A)-Sacular formation in the arterial phase of the study, suggestive of pseudoaneurysm (Arrow), (B)-a microcatheter (inferior arrow) has been advanced superselectively in the lesion arterial branch with Vortex coil (superior arrow).

**Figure 3:**
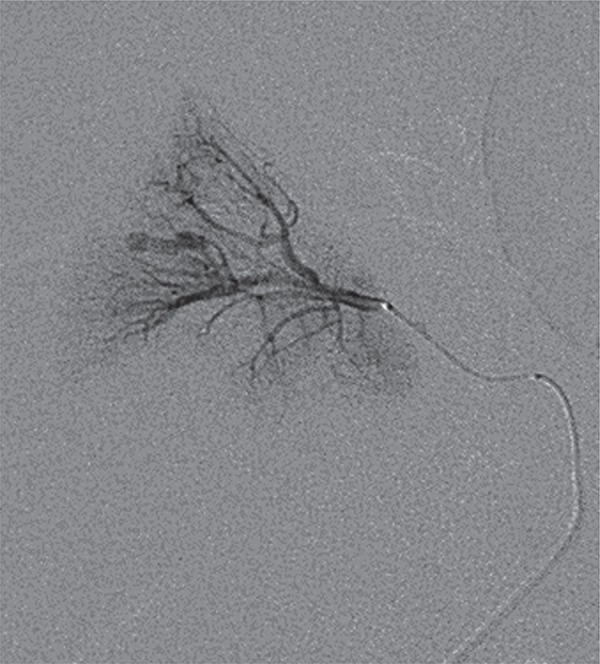
Superselective catheterization of the interlobular artery.

**Figure 4:**
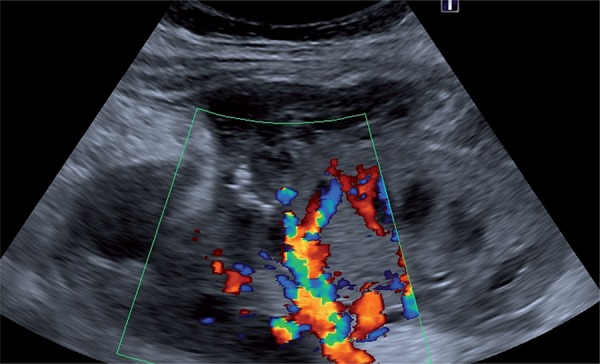
Doppler control demonstrating the closure of the PA.

A CNPB, guided by ultrasonography, was indicated due to delayed graft function at postoperative day 10 which revealed acute tubular necrosis. Shortly after the CNPB she experienced tachycardia, hypotension and decreased blood levels requiring 2 units of blood transfusion and remained stable and developed mild hematuria. An allograft ultrasonography performed five days later revealed an AVF and a PA at the middle pole of the allograft and a peri-renal hematoma around the upper pole with 200cc (Figure-1). A superselective catheterization was then performed, six days after the CNPB with embolization of the PA with two coils. AVF was not observed (Figures 2 and 3). A control by ultrassound 5 days after the procedure assured the closure of the pseudoaneurysm (Figure-4). The patient did well and gradually recovered renal function (creatinine of 1.09mg/dL after two months).
